# A New Algorithm Integrating Molecular Response, Toxicity, and Plasma Level Measures for Ponatinib Dose Choice in Patients Affected by Chronic Myeloid Leukemia

**DOI:** 10.3390/pharmaceutics16030383

**Published:** 2024-03-11

**Authors:** Sara Galimberti, Elisabetta Abruzzese, Giacomo Luci, Claudia Baratè, Luigia Luciano, Alessandra Iurlo, Giovanni Caocci, Riccardo Morganti, Fabio Stefanelli, Antonello Di Paolo

**Affiliations:** 1 Department of Clinical and Experimental Medicine, University of Pisa, 56126 Pisa, Italy; sara.galimberti@unipi.it (S.G.); giacomo.luci@gmail.com (G.L.); 2 Hematology Division, Pisa University Hospital, 56126 Pisa, Italy; claudia.barate@gmail.com (C.B.); r.morganti@ao-pisa.toscana.it (R.M.); 3 Hematology, Sant’Eugenio Hospital, Tor Vergata University, ASL Roma 2, 00133 Rome, Italy; elisabetta.abruzzese@uniroma2.it; 4 Hematology, Federico II University, 80138 Naples, Italy; lulucian@unina.it; 5 Hematology Division, Foundation IRCCS Ca’ Granda Ospedale Maggiore Policlinico, 20122 Milan, Italy; alessandra.iurlo@policlinico.mi.it; 6 Hematology Unit, Businco Hospital, ARNAS Giuseppe Brotzu, 09121 Cagliari, Italy; giovanni.caocci@unica.it; 7 Forensic Toxicology, Pisa University Hospital, 56126 Pisa, Italy; fabio.stefanelli@ao-pisa.toscana.it; 8 Unit of Clinical Pharmacology, Pisa University Hospital, 56126 Pisa, Italy

**Keywords:** chronic myeloid leukemia, ponatinib, therapeutic drug monitoring, molecular response, toxicity

## Abstract

Ponatinib may be effective in chronic myeloid leukemia (CML) patients after failure of first/second line therapies. Although its efficacy for minimum plasma concentrations (C_min_) is >21.3 ng/mL (equal to 40 nM), ponatinib may cause adverse events (AE) that require dose optimization. The present study was aimed at investigating any possible correlations among ponatinib dose, plasma concentration, molecular response (MR), and tolerability in a real-world setting. Clinical and laboratory records (including MR and drug plasma concentrations) of 32 CML patients treated with ponatinib were harvested and analyzed. Twenty-seven patients (71%) had ponatinib C_min_ values > 21.3 ng/mL, but C_min_ values > 10.7 ng/mL (considered efficacious in BCR-Abl unmutated patients) were achieved by 80% of the patients receiving ≥30 mg/day and 45% of the subjects treated with 15 mg/day. No significant correlations were identified among clinical efficacy, tolerability, daily dose, and plasma concentration. Notably, patients who underwent dose tapering for tolerability or safety reasons did not experience treatment failure. In a real-world setting, adjustment of ponatinib daily doses lower than those registered may maintain therapeutic efficacy while reducing the risk of vascular events and improving tolerability. Further studies are warranted to confirm the present results in a larger cohort of patients.

## 1. Introduction

In chronic myeloid leukemia (CML), the balanced reciprocal translocation between the long arms of chromosomes 9 and 22 t(9;22)(q34;q11)—forming the “Philadelphia” (Ph’) chromosome through the creation of the *BCR-ABL1* fusion gene—promotes leukemic stem cell uncontrolled proliferation. By activating multiple signaling pathways, including RAS, RAF, JUN, MYC, and STAT, this fusion gene causes altered cell adhesion, inhibition of apoptosis, differentiation arrest, and proteasomal degradation of several key proteins, leading to the phenotype characterizing Ph’+ disorders [1].

While in the era preceding the introduction of tyrosine kinase inhibitors (TKIs) the only objectives of treatment were containment of leukocytosis and reduction in spleen dimensions, the introduction of TKIs deeply changed the natural history of Ph’+ disorders, as well as the objective of their treatment. Nowadays, if a molecular response of at least 3 logs (MR3) [2] is achieved by the first 6 months of treatment, CML patients’ survival is comparable to that of the general population [3]. Nevertheless, 33% of these patients presented at least one grade 3–4 adverse event, analogously to what was observed in CML, where 20–30% of cases fail to achieve therapeutic goals or develop intolerable side effects or resistance within the first three years of treatment [4,5].

The mechanisms of resistance to TKIs are numerous and still not fully clarified. In 10–15% of cases, the appearance of some *BCR-ABL1* mutations avoids the appropriate attack of TKIs in the ATP-binding pocket [6]. In other cases, the neoplastic cells activate alternative proliferation pathways, such as Beta-catenin/Wnt or JAK-STAT [7], upregulate some genes belonging to epigenetic control, such as Polycomb genes or the PD-L1/PD-1 axis [8,9], or create a hypoxic/hypoglycemic microenvironment that is permissive for leukemic stem cells [10,11]. In addition, polymorphisms of transmembrane drug transporters may also be predictive both regarding TKIs response and poor tolerability [12,13].

To overcome resistance or toxicities to imatinib and second-generation TKIs, other selective and more powerful TKIs have been developed, including ponatinib (PON), efficacious against most BCR-ABL1 mutations, including the “gatekeeper” T315I [14]. In CML, ponatinib offers major cytogenetic responses to 60% and molecular responses to 40% of patients, with a 5-year overall survival of 73% [15].

However, the expected benefits of PON are partially counterbalanced in a third of patients by a broad spectrum of side effects, including high blood pressure, occlusive arterial and venous events, skin rash, myelosuppression, QTc prolongation, and pancreatitis, possibly related to pre-existing cardiovascular risk factors or to administered daily PON dose [16,17,18,19].

Based on in vitro results, arterial occlusive events seem to be dose-related [20], and a post hoc analysis of data derived from three clinical trials on PON in Ph’+ leukemias suggested a 33% reduction in cardio- and cerebrovascular events for each 15 mg decrease in daily PON dose [21]. In the phase-2 OPTIC (Optimizing Ponatinib Treatment in CML) trial, a PON dose reduction to 15 mg with a *BCR-ABL1/ABL1* ratio < 1%IS was obtained [22]. Except for cases with T315I mutations, this dose reduction strategy was successful. On this basis, Castagnetti et al. elaborated on an algorithm helping clinicians regarding PON starting dose choice. In CML patients receiving PON because of poor tolerability, the dose can be established according to the quality of molecular response, reserving higher doses for patients with worse responses, while subjects with optimal responses may start with 30 or 15 mg/day. On the contrary, when PON is chosen because of failure, 45 mg/day must be reserved for patients carrying T315I or compound mutations; warning patients or subjects with high or very high cardiovascular risk can receive 30 mg/day [23].

A recent retrospective analysis performed by Breccia et al. on the Italian prescription registry confirmed clinicians’ concerns about PON-related vascular events risk and their intent to adopt a “de-intensifying” strategy. Among 666 CML patients, 20.4% of them required at least one PON dose reduction due to adverse events, whereas 46.4% had their PON daily dose reduced in the absence of any evidence of side effects as a precaution rather than for a real need [24].

Therefore, it seems clear that, despite the efforts applied in recent years, optimizing PON dose in clinical practice is still an open question, and the present work enters this debate. Indeed, we measured the PON plasma concentrations in 32 CML patients followed in five Italian centers to assess the eventual correlation between PON plasma levels and molecular response or occurring adverse events. Ultimately, we propose a plasma level-oriented algorithm useful for choosing the best PON daily dose.

## 2. Patients and Methods

### 2.1. Study Population

The population enrolled in the study included 32 CML patients treated with PON coming from the Italian Hematology clinics of Pisa, Rome, Milan, Cagliari, and Naples. PON plasma concentrations were evaluated during routine outpatient followup to be non-invasive. Indeed, the PON plasma measures were conducted on plasma remaining from EDTA tubes already used for quantitating the *BCR-ABL1*/*ABL1* ratio, according to the clinical routine. Peripheral blood samples were harvested by the same phlebotomists of each institution during followup visits. Since control visits often occurred after the morning drug intake, the time elapsed from last PON dosing and blood harvest was carefully annotated in the patient’s clinical report for the prediction of minimum plasma values, as already conducted by our group for imatinib [25]. Furthermore, during each visit, physicians registered the fasting status of patient, the use of concomitant drugs, and smoking. The ATNO Ethics Committee granted the authorization of the research protocol (ATNO protocol n. 23707, 27 July 2023). In some cases, the analysis was repeated.

### 2.2. Ponatinib Plasma Concentration Measures

During the followup visits, 5 mL peripheral blood samples were collected in a Lithium Heparin tube. Blood was centrifuged (5 min, 3500 rpm) to separate plasma that was stored at −80 °C until analysis. PON plasma concentrations were determined by High Performance Liquid Chromatography–High Resolution Mass Spectrometry (HPLC–HRMS) by means of a Q-Exactive^TM^ Plus Hybrid Quadrupole-Orbitrap mass spectrometer (Thermo Fisher Scientific, Waltham, MA, USA), associated with a Thermo Scientific UltiMate 3000 HPLC liquid chromatograph (Thermo Fisher Scientific). Standard samples were created from healthy volunteer plasma samples (50 μL each) with a known amount of PON to obtain final concentrations in the range 5–250 ng/mL. The same procedure was performed to obtain quality control samples at a final PON concentration of 25 ng/mL. Deuterated PON was used as internal standard for all samples. Twenty-five μL of plasma samples was directly injected into the HPLC equipped with a purification system TurboFlow On-Line Solid-Phase Extraction (On-Line SPE—Thermo Fisher Scientific). Then, analytes were separated by means of an OMEGA POLAR C18 (Phenomenex, Torrance, CA, USA) in gradient mode with water–formic acid (0.1%, *w*/*v*) and acetonitrile at flow of 0.2 mL/min. Detection of PON and internal standard was performed by HRM positive mode. In these conditions, the method was proven to be linear in the full range of concentrations 5–250 ng/mL, and the limit of quantitation was 5 ng/mL.

### 2.3. Ponatinib Pharmacokinetic Study

To design a PON-specific pharmacokinetic study, we started from the already reported data from Cortes et al., supposing that PON plasma minimum concentration (C_min_) higher than 21.3 ng/mL could be able to inhibit in vitro the selection of new BCR/ABL1-mutated cell clones. Lower concentrations (i.e., 5 ng/mL) could still be effective in inhibiting BCR/ABL1-positive cells’ proliferation but could be less effective against T315I mutated clones [14]. Therefore, measuring PON C_trough_ could help to identify patients at high risk of treatment failure or more prone to develop newly mutated clones. The PON plasma concentration values obtained from our 32 patients on 38 occasions were entered in an electronic database and managed for Population Pharmacokinetics (POP/PK) analysis using Monolix^®^ software version 2021R2 (Lixoft, Antony, France). POP/PK method enables performing complete pharmacokinetics analysis in the absence of a complete series of blood samples, such as those that may derive from carrying out random samplings over time. We adopted a previously published mathematical model consisting of a bi-compartmental model with extravascular absorption (first-order absorption with transit compartments) and first-order elimination (Supplementary Materials) [26,27].

### 2.4. Molecular Response Evaluation and BCR-ABL1 Mutation Analysis

To correlate PON plasma concentrations to the molecular response (MR), quantitative real-time PCR for quantitating the *BCR-ABL1/ABL1* IS% ratio was performed starting from the RNA extracted from 18 mL peripheral blood using the Rotor-Gene^®^ QMDx 5plex HRM and the *BCR-ABL1* Mbcr RGQ RT-PCR Ipsogen Kit^®^. The results were converted according to the International Scale (IS) and standardized according to the national LabNet CML rules (https://www.gimema.it/labnet/labnet-cml/, (accessed on 10 March 2024)).

Mutational analysis of BCR/ABL1 tyrosine kinase domain was performed by using a Next-Generation Sequencing (NGS) platform at GENOMA Molecular Genetics Laboratory Group (Milan, Italy).

### 2.5. Statistical Analysis

Categorical variables were described by frequency, while continuous data by median and range or mean ± standard deviation. The comparison between qualitative variables was performed by chi-squared test, while continuous data were analyzed by *t*-test (two-tailed). To compare qualitative variables with quantitative variables, the Kruskal–Wallis test followed by comparisons with Bonferroni’s inequality or Mann–Whitney tests were used. Spearman’s correlation analysis was performed to compare quantitative variables. Differences were considered statistically significant for *p*-values < 0.05. All analyses were performed by SPSS v.28 software.

## 3. Results

### 3.1. Study Population: Clinical Features and Outcome

The clinical features of the 32 subjects enrolled in the study are reported in Table 1. Seventeen patients received PON as second line, eleven as third line, three as fourth line, and one as sixth line treatment. Overall, eighteen received PON because of failure and fourteen for intolerance to a previous TKI. In two cases, NGS discovered the T315I mutation. A molecular response of 3 logs (MR3) was achieved in 25/38 (65.7%) and deep molecular response (DMR) in 20/38 of cases (52.6%). DMR was never reached by 30% of the patients and was lost by one of them, while MR3 was lost by four patients during the study length. In eight patients who received reduced PON doses, the previously achieved MR level was maintained, and one patient who received a reduced PON dose in a 2-log molecular response (MR2) for adverse events (AE) subsequently achieved DMR.

At the time of data cutoff (November 2023), with a median followup of 27 months, all the patients were alive and in treatment with different PON doses, for a total of 38 measures (PON was administered at 45, 30, and 15 mg/day on 9, 17, and 12 occasions, respectively).

Comprehensively, thirteen toxic events were reported in nine patients, seven at 45 mg/day, four at 30 mg/day, and two at 15 mg/day; in five cases, we observed hematological toxicities (anemia or neutropenia), and, in eight cases, AE concerned extra-hematological toxicity (acute pancreatitis, increased amylase/lipase levels, skin rash, fever, headache, and abdominal pain). Six events were graded as ≥3, with temporary drug interruption and subsequent restart at reduced dose. Two patients definitively interrupted PON for extra-hematological toxicities (arthralgias or pancreatitis). None of the patients experienced cerebro- or cardiovascular events during the study.

### 3.2. PON Plasma Concentrations

In the whole series, the mean PON plasma concentration accounted for 30.19 ± 18.55 ng/mL, and the median value was 27.13 ng/mL (range, 3.16–72.7 ng/mL). In the cohorts receiving 45, 30, and 15 mg/day, the mean values were 41.99 ± 23.77, 34.27 ± 15.19, and 15.25 ± 8.56 ng/mL, respectively, the values in the 15 mg/day cohort being significantly different (*p* < 0.005) from those measured in the other two cohorts. POP/PK analysis was then performed to determine C_min_ and any possible correlation with drug dose. Overall, 27 patients (71%) achieved PON plasma levels ≥ 21.3 ng/mL, while 10 patients (26.3%) reached the target concentration ≥ 10.7 ng/mL. Interestingly, all the patients reached the minimal efficacious concentration (5 ng/mL) regardless of which PON dose they received. On the contrary, concentrations ≥ 10.7 ng/mL were reached by 8/9 patients treated with 45 mg/day (89%), by 14/17 receiving 30 mg/day (82%), and by 5/12 patients receiving 15 mg/day (41.6%) (*p* = 0.038).

**Figure 1:**
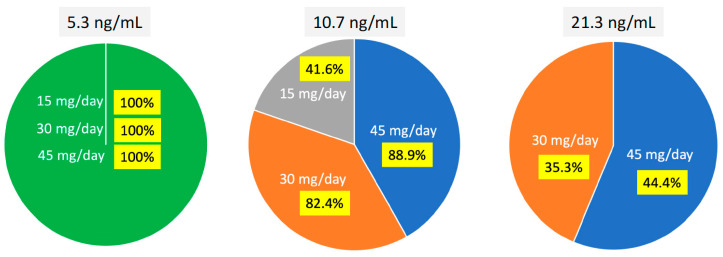
Distribution of patients according to PON dose and threshold values of plasma concentrations of 5.3 (minimum concentration associated with efficacy), 10.7 (efficacy in non-mutated *BCR-Abl* patients), and 21.3 ng/mL (prevention of new *BCR-Abl* mutated clones).

Finally, target concentrations ≥ 21.3 ng/mL were reached by 4/9 patients receiving 45 mg/day (44.4%) and by 6/17 (35.3%) in the group receiving 30 mg/day, but none of the patients treated at 15 mg/day achieved the target (*p* = 0.025) (Figure 1).

Overall, the minimum plasma level of 10.7 ng/mL considered as efficacious in unmutated patients was reached by more than 80% of the patients receiving at least 30 mg/day but also by 45% of the subjects treated with 15 mg/day.

### 3.3. PON Daily Dose, Plasma Concentrations, Molecular Responses, and AE

Further analyses showed that PON daily dose did not condition the quality of MR. Indeed, MR3 was reached by 3/9 patients treated at 45 mg/day (33.3%), by 12/17 cases receiving 30 mg/day (70.6%), and by 10/12 subjects at 15 mg/day (83.3%) (*p* = 0.096). Moreover, DMR was reached by 2/9 patients treated at 45 mg/day (22.2%), by 6/17 cases receiving 30 mg/day (35.3%), and by 7/12 subjects at 15 mg/day (58.3%) (*p* = 0.239). Similar analyses showed that, in the present population, individual PON daily doses were not correlated with occurrence or severity of AE.

When molecular response to PON was investigated based on drug plasma concentration, we did not find significant correlations (Table 2).

Indeed, MR3 was reached by 16/27 (59.3%) and 9/11 (81.8%) of the patients who reached or did not reach 10.7 ng/mL (*p* = 0.268), respectively. In a similar manner, DMR was reached by 10/27 (37%) and 5/11 (45.5%) of the patients who reached or did not reach 10.7 ng/mL (*p* = 0.722), respectively. When we set the threshold at 21.3 ng/mL, MR3 was reached by 6/25 (24%) and 19/25 (76%) of the patients who had PON plasma concentrations higher or lower than 21.3 ng/mL (24%, *p* = 0.709), respectively. Moreover, no significant differences (*p* = 0.473) were observed among the patients who experienced a DMR in terms of PON plasma concentrations greater (5/15 patients, 33.3%) or lower (10/15, 66.7%) than 21.3 ng/mL.

Finally, we tested if PON plasma concentrations might condition the occurrence of AE, but, even in this case, we did not find any significant correlation.

## 4. Discussion

To our knowledge, this study represents the first real-life experience in dosing PON plasma concentrations during routine followup in a cohort of 32 patients affected by CML. In this cohort, PON achieved 65.7% of MR3 and 52.6% of DMR. These percentages are higher than those reported in the pivotal trial PACE [14], but this difference can be explained by some considerations: (1) a different “philosophical” approach by physicians who now use PON earlier than in the past; (2) the difference between the two study cohorts because only 34% of our patients received PON after two other TKIs vs. 93% of the patients enrolled in the PACE study. Analogously, in the PACE trial, 57% of the patients already received three treatments before PON vs. 12% in our cohort. Notwithstanding these differences, even in our real-life multicentric experience, PON was efficacious.

Concerning toxicities, the percentage and quality of AE were superimposable between our series and that from the PACE study (28% vs. 30%), with a discontinuation rate for toxicity of 6% in our series vs. 21% in the pivotal trial. Regarding a previously published larger Italian series of CML patients receiving PON in a real-life scenario [24], the percentages of PON success were similar (65.7% in our series vs. 59% in the registry); note that, in the registry, 39% of the patients received PON as third line treatment compared to 34% in our study.

Dose reduction in the Italian registry was observed in 46% of cases vs. 16% in our study; nevertheless, we must consider that, in our series, we started ab initio with 30 or 15 mg/day in cases that received PON for intolerance but responded favorably.

Noteworthily, this is the largest study that investigated PON plasma concentrations in a clinical setting as only two small similar studies have recently been reported. In 2021, Abumiya et al. described the case of a patient who presented significant hematological toxicities with PON 15 mg/day [28]; two steps of dose reduction (PON 15 mg every other day and then 15 mg every 3 days) and serial PON plasma determinations were performed. The best benefit/risk ratio in this patient was observed with PON 15 mg every 3 days, and the mean plasma concentrations were 35.1 ng/mL at 24 h, 21.1 ng/mL at 48 h, and 14.5 ng/mL at 72 h. MR was maintained, and the hematological toxicity was mild [28].

More recently, Kawano et al. reported a serial determination of PON plasma levels in one patient with chronic-phase CML, one in the advanced phase of CML, and in another patient affected by Ph’+ ALL [29]. All these subjects received PON at low doses: 15 mg every other day or 15 mg/day. Median PON plasma concentrations resulted in 17.2 ng/mL (12.2–34.5 ng/mL), 33.1 ng/mL (21.2–40.3 ng/mL), and 27.7 ng/mL (13.6–29.9 ng/mL). These data are consistent with ours, confirming (1) the possibility to reach the target concentration of 21.3 ng/mL with a PON daily dose lower than the standard dose of 45 mg, and (2) the large intrapatient variability also observed in our cohort. Indeed, in our study, the target concentration of 10.7 ng/mL was reached by more than 80% of the patients treated with 45 or 30 mg/day but also by 45.5% of the subjects receiving 15 mg/day. The threshold of 21.3 ng/mL was reached by at least one third of the patients treated with 45 or 30 mg/day, but none of the subjects receiving 15 mg/day reached that PON concentration.

Those differences are particularly relevant in terms of offering to our patients the best benefit/toxicity ratio. In the OPTIC trial, patients resistant to at least two TKIs or carrying the T315I mutation were randomized to receive PON at 45, 30, or 15 mg/day. Once they reached MR2 or in the case of unacceptable toxicity, the PON dose was reduced to 15 mg/day (in the first two cohorts) or 10 mg/day (in the third cohort) [22]. Noteworthily, 39% of the enrolled patients already received imatinib and another second-generation TKI, and 53% received at least three TKIs, equally distributed in the three groups.

At 12 months, the percentage of patients attaining MR2 accounted for 51.6% in the cohort that reduced the daily dose from 45 mg to 15 mg, while the MR2 rate was lower in the group switching from 30 mg to 15 mg (35.5%) and in patients treated with 15 mg/day (25.3%), with 75% of the patients maintaining MR2. The efficacy rate was very different in patients carrying the T315I mutation because, in the same cohorts described above, the percentages of MR2 were, respectively, 60%, 25%, and 11%.

On the other hand, the discontinuation rates for toxicity were comparable among the three cohorts (19%, 16%, and 14%, respectively), thus demonstrating that the PON daily dose can be adjusted according to MR attainment or the occurrence of AE. Indeed, the need for PON dose reduction derives from the high risk of arterial occlusive events associated with this drug. Based on in vitro data, those events seem to be dose-related, and post hoc analysis suggested a 33% reduction in cardio- and cerebrovascular events rate for each 15 mg decrease in daily PON dose [19].

The results from our study further confirm that PON dose might be adjusted according to the efficacy/toxicity ratio. In our series, the probability of attainment of MR3 or DMR was not significantly conditioned by PON daily dose when 45 mg or 30 mg/day were used. Nevertheless, even with 15 mg/day, we have the possibility of offering a good MR to 40% of our patients, which is a relevant chance for the subjects who do not tolerate PON at higher doses. The pharmacological basis for this phenomenon may be explained by the fact that most of our patients (more than 80%) reach the C_min_ value requested for its efficacy (10.7 ng/mL).

The large inter- and intraindividual variability observed can be explained by different mechanisms, such as drug–drug interactions [30] or polymorphism in membrane transporters or P450 cytochromes [31]. Indeed, in a murine model, drug efflux pumps ABCB1 and ABCG2 markedly reduced the PON brain accumulation, but not its oral availability [32]. Moreover, in a small series of four Japanese patients affected by Ph’+ ALL, the brain distribution of ponatinib was also affected by ABCB1 polymorphisms [33]. Additional factors responsible for the lack of correlations between dose, pharmacokinetics, clinical outcome, and tolerability may include incomplete adherence to the prescription, a well-known phenomenon described in CML patients since the beginning of TKIs use [34,35].

To transfer all these findings into daily clinical practice, the choice regarding the best PON dose might be dynamically based on a combination of the quality of molecular response and the PON plasma levels, either in “resistant” or “intolerant” subjects.

Firstly, we must calculate the cardiovascular risk of our patients by using the Systematic Coronary Risk Evaluation (SCORE2) and SCORE2-Older Persons (SCORE2-OP) scores (https://www.heartscore.org/en_GB/heartscore-europe-risk-regions, (accessed on 10 March 2024)) in European countries or classify subjects according to the American guidelines in the USA and other countries [36].

**Figure 2:**
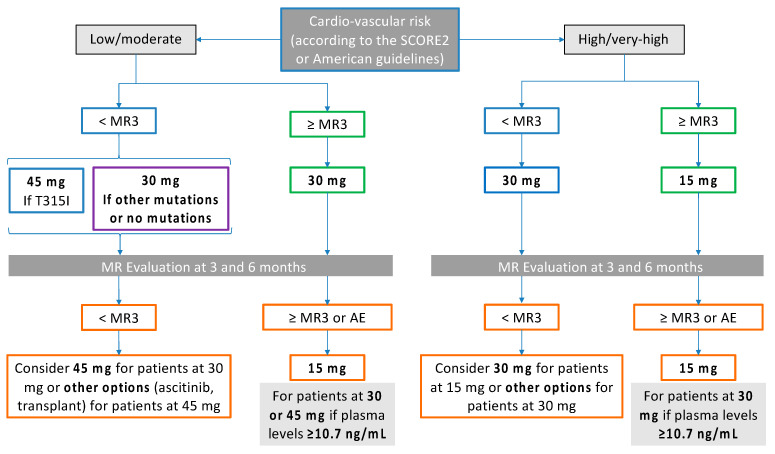
Proposed decisional algorithm for personalized PON dosing in CML patients stratified according to cardiovascular risk and considering the MR and PON plasma levels. Abbreviations: MR, molecular response; MR3, BCR::ABL1/ABL1 ratio ≤ 0.1%.

Indeed, patients at high and very high risk might start PON at 15 mg/day if MR is at least MR3 or at 30 mg/day if MR is lower than MR3. If, after 6 months, a patient at 30 mg/day achieves at least MR3 and PON plasma levels > 10.7 ng/mL, the dose can be reduced to 15 mg, continuing with strict molecular monitoring. On the other hand, patients at low and moderate cardiovascular risk might start PON at 30 mg/day (45 mg/day in the case of demonstrated T315I mutation). After achieving MR3 or with a maintained MR3, the PON dose can be reduced to 15 mg/day if the PON plasma levels are >10.7 ng/mL (see Figure 2).

Our study has some limitations, the first of which is represented by the limited number of patients enrolled. However, those patients came from a real-world setting in which the hematologist pursued ponatinib dose optimization to balance clinical activity and tolerability, and each patient was included in a therapeutic drug monitoring protocol, which is a novelty for ponatinib in this field.

In conclusion, our study performed in a real-world setting demonstrated the feasibility of dose optimization of ponatinib guided by drug monitoring. Based on these results, we propose an algorithm to manage ponatinib in CML patients according to clinical and laboratory findings. Although the algorithm should be validated in a larger population of patients, our results certainly sustain the adoption of such a strategy to improve the clinical management of CML patients, even regarding poor tolerability and efficacy, as started for imatinib [25] and now being conducted for the full spectrum of CML TKIs.

## Figures and Tables

**Table 1:** Characteristics of CML patients enrolled in the present study.

		Number	Percentage
Number of pts		32	/
Sex	Male	16	50%
	Female	16	50%
Age (years)	Median	56.5	/
	Range	22–71	/
Risk score at diagnosis (Sokal)	Low	11	34.4%
	Intermediate	14	43.7%
	High	7	21.9%
Cause of switch to PON	Resistance	18	56.2%
	Toxicity	14	43.8%
Previous lines of treatment	1	17	53.1%
	2	11	34.4%
	>2	4	12.5%
PON daily dose (mg)	45	9	23.7%
	30	17	44.7%
	15	12	31.6%
Best molecular response (38 assessments)	<MR3 ^1^	13	34.2%
	MR3	25	65.7%
	DMR	20	52.6%
Adverse Events (grade 3–4)	Hematological	5	15.6%
	Extra-hematological	8	25%

^1^ MR3, BCR-Abl transcript < 0.1% with respect to basal value; DMR, deep molecular response (i.e., BCR-Abl transcript ≤ 0.01% with respect to basal).

**Table 2:** MR3 and DMR rates among the enrolled patients according to desired threshold values and measured PON minimum plasma concentrations.

Threshold of PON Plasma Concentration	C_min_^1^	MR3	DMR
10.7 ng/mL	Higher than	9/11 (81.8%)	10/27 (37.0%)
	Lower than	16/27 (81.8%)	5/11 (45.5%)
21.3 ng/mL	Higher than	6/25 (24.0%)	5/15 (33.3%)
	Lower than	19/25 (76%)	10/15 (66.6%)

^1^ C_min_, minimum plasma concentrations of PON; MR3, BCR-Abl transcript < 0.1% with respect to basal value; DMR, deep molecular response, BCR-Abl transcript ≤ 0.01% with respect to basal value.

## Data Availability

Data are unavailable due to patients’ privacy. Researchers interested in the present data may contact the corresponding author for further information.

## Supplementary Materials

The following supporting information can be downloaded at: https://www.mdpi.com/article/10.3390/pharmaceutics16030383/s1, Figure S1: Measured PON concentrations vs. population (left) and individual prediction values (right); Figure S2: Scatter distribution of individual weighted residuals (iWRES) with respect to time (left) and individual predictions (right); Figure S3: Probability (left) and cumulative (right) distribution of individual weighted residuals (iWRES); Figure S4: Prediction-corrected visual predictive check (pcVPC) of present data; Table S1: Values of pharmacokinetic parameters [26] and the corresponding values in the actual population of 32 CML patients.
