# Supplementary material for: A New Algorithm Integrating Molecular Response, Toxicity, and Plasma Level Measures for Ponatinib Dose Choice in Patients Affected by Chronic Myeloid Leukemia
Source: Pharmaceutics. 2024 Mar 11;16(3):383. doi: 10.3390/pharmaceutics16030383 (PMC10975223; doi:10.3390/pharmaceutics16030383)
Supplement: Supplementary file 1 [file pharmaceutics-16-00383-s001.zip › pharmaceutics-2821020-supplementary.pdf]

A new algorithm integrating molecular response, toxicity and plasma levels measure for the ponatinib dose choice in patients affected by chronic myeloid leukemia

Sara Galimberti et al.

Supplemental data - <u>Pharmacometric analysis and results</u>

Population Pharmacokinetics (POP/PK) analysis was performed using Monolix ® software vers. 2021R2 (Lixoft, Antony, France) on 32 enrolled patients with 38 measurements of PON plasma concentrations. A previously published mathematical model consisting of a bi-compartmental model with extravascular absorption (first-order absorption with transit compartments) and first-order elimination was employed [26]. Due to the reduced number of patients and observations, and the major impact of the absorption process through transit compartments rather than the lag-time absorption process [26], the absorption of PON was considered only by transit compartments with an initial estimate of mean transit time (*Mtt*) equal to 3.072 based on the value of k_a (equal to k_{tr}) according to the following formula:

$$Mtt = \frac{(n+1)}{k_{tr}}$$

where n is the number of transit compartment [27]. Initial and final values of PK parameters are presented in the following Table S1.

Table S1. Values of pharmacokinetic parameters [26] and the corresponding values in the actual population of 32 CML patients

PK parameter	Initial values		Final values	
	Value	RSE(%)	Value	RSE(%)
k_a[1] (= k_{tr}, h^{-1})	1.302	94.4%	0.979	108.44
**Mtt** (h)	3.072*	-	4.525	24.13
**CL/F** (L/h)	34.28	3.23	29.24	16.11
V_{centr}**/F** (L)	838.6	3.56	746.59	26.81
**Q/F** (L/h)	17.21	7.33	15.83	10.54
V_{per}**/F** (L)	347.4	5.25	435.25	8.37
**Residual variability (%)**	38.59	20.1	37.97	34.50
**Interindividual variability**				
k_a	46.85%	6.84	55.77%	15.28
**Mtt**	-	-	29.72%	34.94
**CL/F**	48.04%	6.03	51.42%	24.37
V_{centr}**/F**	42.33%	9.88	66.52%	15.12

[1]k_a absorption constant; Mtt, mean transit time; CL/F and Q/F, apparent systemic and intercompartmental clearance, respectively; V_{centr}/F and V_{per}/F, apparent volumes of central and peripheral compartments, respectively; BW, body weight; RSE, relative standard error; *, initial value obtained as described in the text

Largest RSE% values were likely due to the limited number of patients enrolled in the present population. However, diagnostic plots show good fitting of the measured PON plasma concentrations without evident error trends (Figures S1-S4).

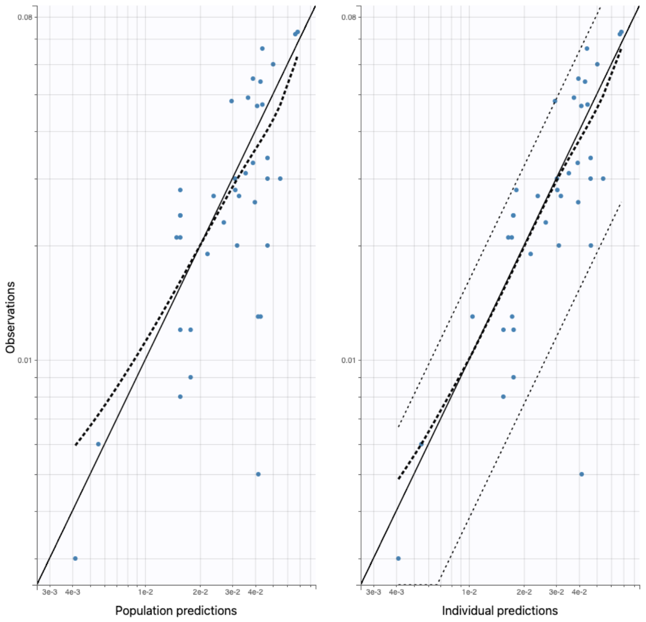

Figure S1. Measured PON concentrations vs. population (left) and individual prediction values (right). Symbols, observed values; continuous line, line of identity; thin dashed lines, 90% confidence interval; thick dashed line, spline.

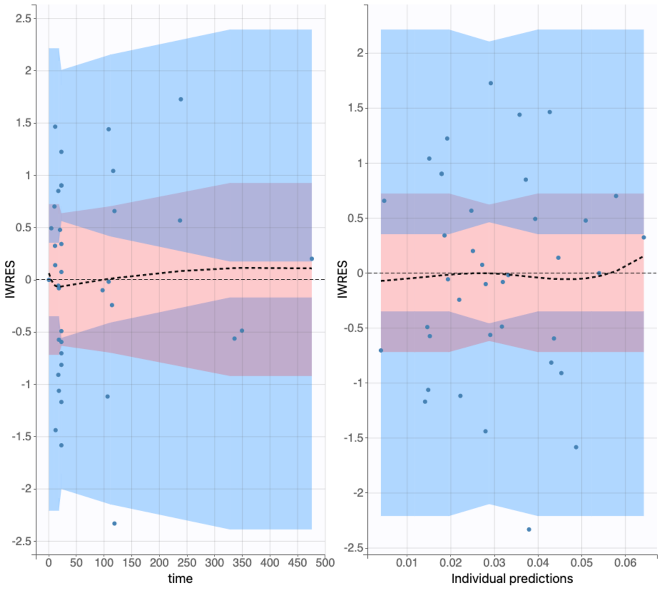

Figure S2. Scatter distribution of individual weighted residuals (iWRES) with respect to time (left) and individual predictions (right). Symbols, observed values; shaded areas, 90% confidence intervals of 5th, 50th, and 95th percentile of the actual distribution; black dashed line, spline.

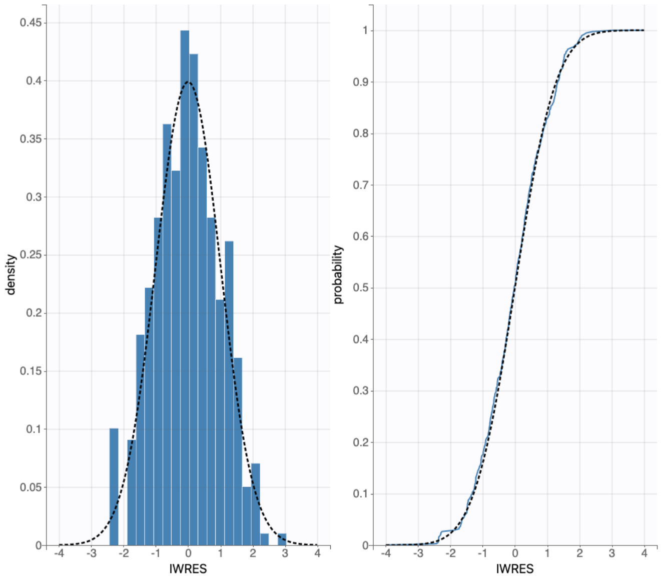

Figure S3. Probability (left) and cumulative (right) distribution of individual weighted residuals (iWRES). Black dashed line, theoretical distribution; bars and continuous blue line, empirical distirbution.

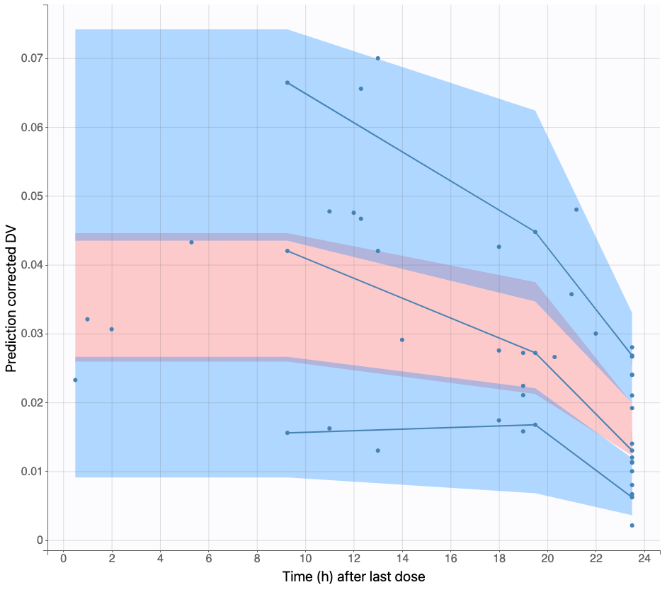

Figure S4. Prediction-corrected visual predictive check (pcVPC) of present data. Symbols, observed values; continuous lines, 5th, 50th, and 95th percentiles of the actual distribution; shaded areas, 90% confidence intervals of the predicted 5th, 50th, and 95th percentiles.

References

26. Hanley, M.J.; Diderichsen, P.M.; Narasimhan, N.; Srivastava, S.; Gupta, N.; Venkatakrishnan, K. Population Pharmacokinetics of Ponatinib in Healthy Adult Volunteers and Patients With Hematologic Malignancies and Model-Informed Dose Selection for Pediatric Development. *J. Clin. Pharmacol.* **2022**, *62*, 555–567, doi:10.1002/jcph.1990.
27. Savic, R.M.; Jonker, D.M.; Kerbusch, T.; Karlsson, M.O. Implementation of a transit compartment model for describing drug absorption in pharmacokinetic studies. *J. Pharmacokinet. Pharmacodyn.* **2007**, 34, 711-726. doi: 10.1007/s10928-007-9066-0.
